# Morpho Evo-Devo of the Gynoecium: Heterotopy, Redefinition of the Carpel, and a Topographic Approach

**DOI:** 10.3390/plants13050599

**Published:** 2024-02-22

**Authors:** Rolf Sattler

**Affiliations:** Biology Department, McGill University, Montreal, QC H3A 0G4, Canada; sattler.rolf@gmail.com

**Keywords:** plant evo-devo, plant morpho evo-devo, gynoecium, carpel, gynoecial appendage, spatial shifting, heterotopy, topographic approach, fossil angiosperms and pre-angiosperms, flower concept, angiosperm evolution

## Abstract

Since the 19th century, we have had countless debates, sometimes acrimonious, about the nature of the gynoecium. A pivotal question has been whether all angiosperms possess carpels or if some or all angiosperms are acarpellate. We can resolve these debates if we do not define the carpel as a closed megasporophyll but simply as an appendage that encloses the placenta or a single ovule. This redefinition may, however, lead to confusion because often it may not be clear whether the traditional (classical) definition of the carpel or the redefinition is implied. Therefore, a topographic approach is proposed that is compatible with the redefinition. According to this approach, gynoecia comprise one or more gynoecial appendages and placentas or single ovules that may be formed in different positions. Heterotopy refers to these different positions. In the context of evo-devo, which explores evolutionary changes in development, morpho evo-devo delves into spatial shifts of the placentas and ovules leading to heterotopy. Furthermore, it considers shifts in timing (heterochrony) and other processes leading to heteromorphy. Recognizing spatial shifting of the placentas or a single ovule and other evolutionary processes opens up new vistas in the search for the ancestor(s) of angiosperms and their gynoecia.

## 1. Introduction

Plant evo-devo investigates the evolution of plant development. It integrates evolutionary theory, morphology, and molecular genetics. Morpho evo-devo emphasizes the morphological aspects of evo-devo [1,2,3,4]. Thus, in this article, the focus is on the morphological evolutionary changes in the development of the gynoecium. According to classical mainstream thinking, the gynoecium consists of carpels. However, the carpel concept can be unambiguously applied only to a limited extent. I shall, therefore, present a redefinition of the carpel that applies to a much greater extent; and in line with and as an elaboration of this redefinition, I shall propose a topographic approach to the gynoecium that overcomes problems and pseudo-problems in gynoecial morphology. Heterotopy (different positions) of ovules and placentas, due to spatial shifting, plays a crucial role in the topographic approach, in morpho evo-devo, and in the search for the ancestral condition of the gynoecium.

## 2. Definition of the Carpel

Classical plant morphology originated in the 18th century. Goethe’s *Metamorphosis of Plants* [5] is one of its cornerstones. Subsequently, it has been embraced by many botanists, including Troll and Kaplan who have been very influential (for a historical review see, for example, Endress [6]). According to classical morphology, a carpel is a phyllome (leaf homologue) that bears and encloses ovule(s); hence, it has been considered a closed megasporophyll (see, for example, [6,7]). Although this definition dominates mainstream plant morphology, it has been contested since the 19th century. Even Goethe himself was critical. At the end of his life, in *Prose Maxims* (published posthumously), he said: “The pistil, the receptacle, and the fruit all belong to the system of eyes [i.e., branches]” (quoted by Cusset [8] (p. 27). Hence, he denied the existence of classical carpels, which earlier in his life he had postulated in his *Metamorphosis of Plants* [5]. Subsequently, other morphologists proposed alternative views [9]. Brückner [9] distinguished ten different interpretations of the carpel or gynoecial unit. Lorch [10], in a historical overview of the carpel concept, concluded that “even on its own, classical morphology upon advancing toward smaller and less obvious targets would have found itself confronted with the breakdown of the concept of the carpel”. Thus, first I want to examine problems and pseudo-problems with the classical carpel concept.

## 3. Do All Gynoecia Have Carpels?

According to mainstream thinking (classical morphology), one of the defining characteristics of angiosperms is the possession of classical carpels (leaf homologues). Hence, all gynoecia in angiosperms are considered carpellate. However, based on developmental studies, this claim has been contradicted or has at least become questionable. Of course, the gynoecia of many taxa of angiosperms have carpels if carpels are defined as appendages of the gynoecium (referred to as gynoecial appendages) that bear and enclose ovule(s). However, it is important to note that such carpels should not be classified solely as megasporopylls (phyllomes), as they go beyond being mere phyllomes—they are phyllomes that bear ovules; hence, they are composite structures. Nevertheless, in a significant number of taxa, it is difficult or impossible to apply this carpel concept of a composite structure or the classical phyllomic concept (see, for example, [11]). Developmental investigations indicate that in these cases, the centre of the gynoecium is transformed into a single ovule or a placenta that bears the ovules (see, for example, [11,12]), or an ovule is formed in the axil of gynoecial primordia [13,14]. Nonetheless, classical morphologists such as Endress [6] and Kaplan [7] insist that all gynoecia have carpels. Endress [6] claimed that in gynoecia with a terminal ovule or placenta (where the ovule or placenta arises in the centre of the gynoecium), the young carpels “are ‘rooted’ within the remaining floral apex”. How, then, can he delimit these carpellary “roots” from the floral apex? It seems difficult or impossible to draw a line between the floral apex and carpel primordia. Enquiring about the limit of primordia where none exists in nature seems to be a pseudo-problem. To avoid this pseudo-problem, one could postulate a congenital fusion of the base of the carpel primordium with underlying tissue and the floral apex so that carpellary tissue would cover the floral apex [15]. But congenital fusion is a “fusion”, that, by definition, is in principle unobservable because it does not involve any observable fusion. Hence, the admission of this concept removes the empirical basis of morphology. Furthermore, if a central terminal ovule is claimed to belong to a carpel, the question would be to which one when more than one is present. For example, in *Myrica gale*, the gynoecium has two gynoecial appendages and one terminal basal ovule [12]. If these two gynoecial appendages are interpreted as carpels, then the ovule should belong to one of them, should it not? However, Sokoloff et al. [16], referring to mixomery, thought that an ovule could be shared between carpels.

In any case, concerning the concept of congenital fusion, from the perspective of morpho evo-devo, the challenge is to discover the observable developmental processes that are subsumed and often hidden by the concept of congenital fusion. Sattler [17] distinguished the following processes: zonal growth, heterotopy, meristem extension, and interprimordial growth. Sokoloff et al. [18] (p. 18) equate congenital fusion with zonal growth, which is only one of the processes subsumed under the concept of congenital fusion. But note that zonal growth does not imply any kind of fusion. Heterotopy also does not involve any kind of fusion and neither does meristem extension. Interprimordial growth leads to continuity between primordia such as the gynoecial primordia. It involves an observable fusion process only if it occurs after the inception of adjacent primordia, in which case it is not subsumed under congenital fusion but represents meristem fusion. Thus, none of the processes subsumed under the concept of congenital fusion constitute a fusion process. However, further clarification of developmental processes subsumed under the term congenital fusion appears desirable. In any case, for morpho evo-devo, the basis must be the elucidation of developmental processes that change during evolution and that are at least in principle observable. If this condition is not fulfilled, morpho evo-devo and morphology lose their empirical basis. I aim to place morpho evo-devo and morphology on an empirical basis.

One might avoid the problem of congenital fusion by assuming that as a result of the formation of gynoecial primordia, the floral apex is used up and thus disappears so that the base of the gyoecial primordia extends into the centre of the gynoecium. However, in taxa such as *Basella rubra* [11], the centre of the gynoecium retains the organization of a typical floral apex, which then is gradually transformed into an ovule. Hence, the gynoecium has been considered acarpellate. Nonetheless, the controversy about acarpellate gynoecia continues (see, for example, [6,19]). But a redefinition of the carpel and a topographic approach can end and supersede this longstanding controversy.

## 4. Redefining the Carpel

Instead of concluding that there are carpellate and acarpellate gynoecia, an alternative would be a redefinition of the carpel concept in such a way that most of the acarpellate or questionable gynoecia would become carpellate. Thus, according to one redefinition, the carpel is a gynoecial appendage that encloses ovule(s) but does not necessarily bear them [11,20,21] (in their Glossary only). For example, with regard to Caryophyllales, Ronse De Craene [22] pointed out a “progressive detachment of ovules from the carpellary tissue”. Also in the Caryophyllales, Cresens and Smets [23] referred to “topographically cauline placentation”.

Although this redefinition of the carpel concept is broader than the classical carpel concept, it still has a potential weakness, because in any particular case, someone might ask whether the ovule(s) are borne on the carpel or the floral apex. How do we answer this question? We would have to delimit the carpel primordium from the floral apex. But, as pointed out already, it seems difficult or impossible to delimit growth centres such as the carpel primordium and the floral apex. Therefore, it may be difficult or impossible to decide whether ovule(s) are borne on the carpel or the floral apex. To overcome this problem, which may be a pseudo-problem, we proposed to redefine a carpel simply “as an appendage which ENCLOSES ovule(s)” [24] (p. 181). The ovule(s) may be enclosed by one or more than one appendage. Furthermore, the appendages may be interconnected due to interprimordial growth, which in extreme cases, leads to a ring primordium. The enclosure may not always be complete and may be achieved in different ways [25]. It may also involve growth underneath the appendages. However, the redefinition does not require difficult or impossible delimitations; therefore, I consider it the most appropriate redefinition. When I refer to redefinition, this one is meant.

One could give a new name to this redefinition such as ‘gynomer’, as one anonymous reviewer suggested. But it may be difficult to have such a new name widely accepted. On the other hand, if we continue using the term carpel, it may lead to confusion unless we always specify whether the term is used in the classical sense, as a composite structure, as redefined simply as an enclosing structure, or in yet different ways [9]. Therefore, instead of the term carpel, I propose using the terms gynoecial appendage and gynoecial primordium, which do not require further specification. In my book *Organogenesis of Flowers* [26], I consistently used these terms. Referring to gynoecial appendages and gynoecial primordia does not imply whether an ovule or placenta is borne on them. These terms specify only the formation of a primordium that develops into an appendage, which is an observable phenomenon. The formation of a placenta or an ovule is then an independent event. The topographic approach is predicated on this distinction of the formation of a gynoecial appendage on the one hand and a placenta or ovule on the other. Croizat [27] already emphasized the importance of this distinction. And Santos and Wang [28] referred to the Unifying Theory [29], according to which the carpel is “a composite organ comprising two parts of different nature”. Vasculature has also been used as supporting evidence for this notion [30].

## 5. A Topographic Approach to the Gynoecium

The development of the gynoecium begins with the initiation of one or more gynoecial primordia. Subsequently, placentas or single ovules are formed in various positions. The topographic approach specifies the topography, that is, the position of placentas or single ovules in relation to the gynoecial primordia that develop into gynoecial appendages. Thus, in this approach, the question is no longer whether a placenta or ovule is formed on the floral apex or not. What matters is only their position. We can distinguish the following positions: basal or central, axillary, and appendicular; and for the latter, submarginal, ventral, dorsal, and laminar; and, for syncarpous gynoecia, free central, axile, parietal, and superficial. Aberrant and intermediate positions can also be recognized such as, for example, a near-basal ovular position in taxa such as *Hordeum vulgare* [26]. As a result, a continuum of positions can be envisaged.

This topographic terminology has been used by taxonomists for a long time and seems to work well. However, for many morphologists, this approach might entail an evasion of interpretation. They would consider the proposed topographic approach to be only descriptive. However, some philosophers, including Nietzsche, have pointed out that all descriptions are interpretations because all descriptions involve concepts that interpret them. Thus, the proposed topographic approach can be seen as a topographic interpretation of the gynoecium, based on observable phenomena.

One great advantage of the topographic approach is that it allows adherents and defenders of opposite views, the carpellate versus the cauline interpretation of the gynoecium, to meet and shake hands because they can agree about the position of placentas and ovules: they can agree that in free central and basal placentation, the placenta or ovule arise in the centre of the gynoecium. Furthermore, understanding processes such as spatial shifting that leads to heterotopy of the placenta or ovule can be seen as more basic than the structural categories of caulome and phyllome. This view has been elaborated in process morphology that is beyond structural categories [31].

The topographic approach overcomes futile debates that have persisted since the 19th century. It can redirect research toward more productive avenues since it can liberate us from the fruitless search for boundaries that do not exist in nature such as the boundaries of gynoecial primordia. Thus, the distinction between carpellate and acarpellate gynoecia, phyllospory and stachyospory [32] is transcended (see also [33]). However, when placentation is clearly appendicular, the classical carpel concept is still applicable and useful. But even then it is important to recognize that the carpel is not just a phyllome but a composite structure consisting of a more or less foliaceous appendage and a placenta or single ovule.

(As an autobiographical note, I might mention that my morphological thinking about the gynoecium evolved from an acceptance of the classical carpel concept in my doctoral thesis [34], to a distinction of carpellate and acarpellate gynoecia [15], to a redefinition of the carpel [24], and finally, in this article, to a topographic approach that supersedes longstanding futile controversies and pseudo-problems.)

## 6. Morpho Evo-Devo of the Gynoecium

The topographic approach provides an empirical basis for morpho evo-devo research of the gynoecium. This empirical basis encompasses the morphological development of gynoecia, making the exploration of gynoecial development fundamental. It is through this elucidation that we can then pose questions about the transformation of diverse gynoecia into one another during evolution. Notably, we discover that this transformation occurred through many processes. One of them is spatial shifting, which implies heterotopy, especially heterotopy of the placenta or a single ovule. As a result of heterotopy, the placenta or a single ovule may arise on the gynoecial appendages, which leads to classical carpels, or in the axil of the gynoecial appendages, or in the centre of the gynoecium. From a topographic perspective, we would simply say that the placenta or the single ovule may be appendicular, axillary, or central (basal). Furthermore, a continuum of these positions may be envisaged. A single axillary ovule occurs in *Illicium lanceolatum* [13], *Illicium henryi* [35], and *Ochna atropurpurea* [14]. An ovule-bearing branch is formed in the axil of gynoecial appendages of atypical gynoecia of *Michelia figo* [36]. In *Myrica gale* [12] and *Basella rubra* [11], a single ovule is formed in the centre of the gynoecium, which means the ovule is basal. Sattler and Lacroix [11] (p. 926) listed many other taxa in which the ovule is basal or the placenta is free central (for a more complete list contact Prof. Christian Lacroix, Biology Dept., University of Prince Edward Island, Charlottetown, P. E. I.).

According to mainstream thinking, appendicular placentation is considered primitive, whereas axillary and basal or central placentation would be derived. The latter could have evolved independently several times. For example, according to Ronse De Craene [22], even within the Caryophyllales, a basal ovule evolved independently several times. Nonetheless, one cannot exclude the possibility of evolution in the opposite direction in at least some taxa. And one cannot exclude the possibility that axillary, basal or central placentation may have occurred in early angiosperms or pre-angiosperms, subsequently leading directly to these placentation types in at least some taxa of extant angiosperms. The current molecular phylogenetic framework does not support this view [37,38], but the discovery of fossil angiosperms and pre-angiosperms may lead to new insights in this respect.

## 7. Gynoecia of Fossil Angiosperms and Pre-Angiosperms

The recognition of various positions of the placenta or a single ovule is not only important for an understanding of gynoecial morphology in extant angiosperms but is also highly relevant for the search for fossil angiosperms and pre-angiosperms. As long as we are blinded by the assumption that all angiosperms have classical carpels, we are likely to look for fossils whose gynoecia resemble classical carpels at least in some ways. However, bearing in mind the diversity of placentation and ovular position, we are not locked into this view; we can also envisage other possibilities. Thus, *Combina* gen. nov., discovered in the middle Triassic by Santos and Wang [28], can be considered a precursor of angiosperms. *Combina* has an axillary ovule that is almost fully enveloped by a bract. This condition resembles the one reported in taxa such as *Illicium* as noted above. If this condition is ancestral to the angiospermous gynoecium, then classical carpels would have evolved through shifting (heterotopy) of the ovule onto the margin of the gynoecial appendage and an increase in its number as it can be seen, for example, in a pea pod. Having many ovules instead of only one axillary one could be seen as advantageous.

Santos and Wang [28] (Figure 4) proposed an evolutionary trend leading from the fossil *Drepanolepis*, whose ovule is in the axil of a bract that does not enclose it, to *Combina* where the bract almost completely encloses the ovule, and then to taxa like *Illicium* where the ovule is fully enclosed. If this hypothesis can be confirmed, the question remains whether this was the only way the gynoecium of angiosperms originated or whether additional ways existed, as proposed by some authors (see, for example, [27,33,39,40] and below). Wang [33] concluded: “New knowledge of angiosperms, fossil and extant, seems to suggest that the multiphyly of angiosperms cannot be excluded from the alternative list for the time being”.

In any case, angiosperms have their placenta and ovule(s) enclosed. This enclosure might have evolved in different ways. Wang [33] referred to a great diversity of ovule-enclosing ways in angiosperms. *Archaefructus*, a fossil angiosperm from the early Cretaceous [41], has carpels, but they are unusual because the ovules are inserted dorsally along the midrib of the carpel [29], a condition also known in extant angiosperms such as in Cabombaceae. In *Archaefructus*, the number of ovules varies from one to twelve [29]. If there is only one ovule, where exactly is it positioned? If it is positioned at or near the axil of the carpel, it would be similar to the position of the axillary ovule of *Combina* and *Illicium*. Then, starting with the axillary ovule of *Combina*, the carpel of *Archaefructus* could have evolved through an amplification of the number of ovules along the midrib of the carpel. Subsequently, spatial shifting of ovule formation from the midrib towards the margins could have produced the conduplicate angiospermous carpel such as the pea pod. Furthermore, spatial shifting of axillary ovule formation into a ventral median position of an ascidiate carpel could have led to gynoecia of primitive angiosperms such as that of *Amborella*. Further shifting into the centre of the gynoecium could have produced the basal ovule in gynoecia such as that of *Myrica*. However, the basal ovule in *Myrica* and other taxa might have been primitive, although this view is not supported in the current molecular phylogenetic framework. The Middle Jurassic *Qingganninginfructus formosa* had a single basal bitegmic ovule [42]. Hence, in the Jurassic, we find already basal ovules. Furthermore, the Triassic *Nubilora*, “although not a bona fide angiosperm” [43], had ovules directly borne on the floral axis [28,29]. The “flower” of the Jurassic *Nanjinganthus* had an inferior ovary [44,45]. However, like the other Jurassic fossils, it remains controversial [37,38].

Several alternative hypotheses concerning the origin of angiosperms and their gynoecia have been proposed (see, for example, [12,46,47,48]. However, all of them remain controversial. While the present proposal seems more supported by the fossil record, it is also hypothetical and speculative. Nevertheless, we can at least conclude that heterotopy of placentas and ovules occurred in both extant and fossil angiosperms. This heterotopy includes appendicular, axillary, and basal or central positions. The latter two appear to be most easily understood through a topographic approach or at least through a redefinition of the carpel if one wants to retain this term; but, as I suggested, the term “gynoecial appendage” would be preferable. In any case, understanding developmental and evolutionary processes is fundamentally important.

## 8. Processes in Morpho Evo-Devo of the Gynoecium

Since morpho evo-devo investigates developmental changes in evolution, it involves the processes underlying these developmental changes. As pointed out above, one important process is spatial shifting (leading to heterotopy). Within extant and fossil angiosperms, positional shifts may have occurred in various directions [11]. Once the placenta or a single ovule has been enclosed, such shifts would not endanger the survival of such species.

Besides spatial shifting (heterotopy), another process is temporal shifting (heterochrony). For example, in the gynoecium of grasses, a heterochronous shift in ovule initiation was reported [49]. An extreme example of heterochrony occurs in *Balanophora elongata* where the embryo sac develops already within an elongate floral apex [50] (p. 531). Hence, no gynoecial appendages and ovules are formed.

In addition to heterotopy and heterochrony, Zimmermann [50] referred to heteromorphy. The following processes leading to heteromorphy played an important role in morpho evo-devo of the gynoecium:

1. Differentiation, such as the differentiation of the gynoecial appendages into ovary, style, and stigma.
2. Varying proportions, such as the varying proportions of the ovary, style, stigma, and other components. (e.g., [51]).
3. Zonal growth and interprimordial growth [17]. Zonal growth can produce inferior ovaries and cup-shaped invaginations in flowers such as *Amborella* [52].
4. Postgenital fusions (surface fusions) between various organs. In contrast to congenital fusion, which usually is in principle unobservable, postgenital fusion is observable.
5. Reduction or amplification in size and number, as, for example, the size of the gynoecial appendages and the number of ovules.
6. Transference of function [53]. For example, in *Stylidium adnatum* the function of the style has been transferred to the androecial tube [15,26].

Takhtajan [54], like Zimmermann [50], also a forerunner of plant morpho evo-devo, distinguished the processes of deviation, prolongation, and abbreviation. Heterotopy and the above processes (# 1–6) would be examples of deviation, whereas prolongation and abbreviation would be examples of heterochrony.

Considering all relevant processes provides a more complete picture of morpho evo-devo of the gynoecium.

## 9. Morpho Evo-Devo and the Concept of the Flower

Morpho evo-devo of the gynoecium is relevant to the concept of the flower. According to the predominant classical morphology, “a flower is a reproductive short shoot bearing microsporophylls (stamens)… and megasporophylls (carpels) as its appendages or leaf homologues” [7] (p. 1069, edited by P. K. Endress). Given the available developmental data, this definition is no longer generally valid. The appendages of the androecium range from leaf-like to stem-like and even short shoot-like structures [55,56]. And it is questionable whether all gynoecia consist of closed megasporophylls. How, then, can we define the flower?

According to Claßen-Bockhoff [57], the flower is the “sporangia bearing tip of the shoot”, in which “stamens and carpels are sporangiophores and as such ‘de novo’ structures not necessarily homologous with vegetative leaves”. Whereas one can see the androecium as consisting of sporangiophores that may be more or less leaf-like, stem-like, short shoot-like, or do not fit any of the classical categories, the gynoecium is more complex, consisting of more or less leaf-like appendages (gynoecial appendages) that enclose the sporangiophores, which in the simplest case, consist only of one ovule and in more elaborate cases, of a placenta with ovules. As pointed out above, the position of the placentas and ovules is variable. In gynoecia with classical carpels, the sporangiophores are formed on the carpels, whereas in other gynoecia, for example, in *Myrica*, they are basal, or as, for example, in *Illicium*, they are axillary. Alternatively, one could consider the gynoecial sporangiophore as a dual structure consisting of a more or less foliaceous appendage and a sporangia-bearing structure (see also Claßen-Bockhoff’s magnum opus [58]).

According to process morphology, stamens and carpels, gynoecial appendages, placentas, and ovules are seen as process combinations that need not be fitted into the classical categories of stem and leaf [31]. These process combinations may change during evolution and shift their positions leading to heterotopy.

## 10. Conclusions

Morpho evo-devo should be based on observable developmental changes and the processes that generate these changes during development and evolution. Spatial shifting, leading to heterotopy, is a basic process that gave rise to gynoecia with ovules formed in different positions: on the gynoecial appendage or in its axil or the centre of the gynoecium. In the latter case, ovules are not “as if glued on the surface of the gynoecium or the floral apex”, as Endress [6] misrepresented our investigations; but the floral apex, retaining first its typical organization, is gradually transformed into a single ovule or a placenta (see, for example, [11] (Figures 12–15)). From the topographic perspective, one would not even refer to the floral apex, but simply to a basal ovule or a free central placenta. This view has an empirical basis; it does not require difficult or impossible delimitations of gynoecial primordia that have led to almost endless futile controversies since the 19th century. This view supersedes the either/or question: is placentation cauline or phyllomic? Instead, we ask questions about the role of heterotopy in development and evolution. These questions appear more productive. Sometimes progress is made in science by asking new or different questions. Questions about boundaries of primordia that do not exist in nature are pseudo-questions, which are an obstacle to progress.

In addition to spatial shifting (heterotopy), temporal shifting (heterochrony), differentiation, varying proportions, interprimordial and zonal growth, reduction, amplification, and transference of function are processes that played an important role in morpho evo-devo of the gynoecium.

As a result of a morpho evo-devo perspective based on developmental studies of gynoecia, angiosperms cannot be defined by the possession of classical carpels (closed megasporophylls), although many taxa have such carpels. As the name angiosperms indicates, they are defined by having the ovules and seeds enclosed, enclosed by gynoecial appendages and in many cases, by additional underlying tissue formed by zonal growth, as, for example, in inferior ovaries. Within the enclosure and protected by it, placentas and ovules could change their position without any detrimental effect on the survival of the plants.

In this article, the focus has been on morpho evo-devo. This focus will have to be enlarged to include data from developmental genetics (see, for example, [59,60,61,62,63,64]). For the carpel, Mathews and Kramer [64] concluded “that the carpel is a complex organ consisting of a foliaceous appendage and the placenta”. When this is recognized that the carpel consists of two components, the foliaceous appendage (which I call the gynoecial appendage) and the placenta or a single ovule, then we can also recognize that the relative position of the two components may change and that this change may eclipse the classical carpel when the placenta or ovule is free central, basal, or axillary. Change in the relative position may be related to the notion of ectopic gene expression.

Recognizing that in extant angiosperms ovules are not always appendicular is also relevant for the search of fossil angiosperms and pre-angiosperms. It opens up different vistas in the search for angiosperm ancestor(s). Thus, the Triassic *Combina* can be considered a possible ancestor of angiosperms. It has bracts that almost completely enclose an axillary ovule. Spatial shifting of axillary ovule formation into a ventral median position of an ascidiate carpel could have led to gynoecia of primitive angiosperms such as that of *Amborella*. Spatial shifting of ovule formation into the midrib region of the enclosing appendage and an increase in the number of ovules could have produced the carpels of the early Cretaceous fossil *Archaefructus*. And spatial shifting of its ovules toward the margins of the gynoecial appendage could have led to the conduplicate carpel (folded megasporophyll) of extant angiosperms such as that of Magnoliaceae. If we assume a monophyletic origin of angiosperms, further shifting of ovule formation into the centre of the gynoecium could have produced gynoecia such as that of *Myrica* in which the ovule is basal. However, basal ovules as in *Myrica* might also be primitive since there is evidence of basal ovules already in the Triassic and Jurassic.

Although the origin and evolution of angiosperms and the gynoecium remain hypothetical and speculative, we can at least conclude that heterotopy of placentas and ovules occurred in both extant and fossil angiosperms. This heterotopy includes appendicular, axillary, and basal or central positions. The latter two appear to be most easily understood through a topographic approach or at least through a redefinition of the carpel, if one wants to retain this term; but, as I suggested, the term gynoecial appendage would be preferable.

According to process morphology, carpels, gynoecial appendages, placentas, and ovules are seen as process combinations that need not be fitted into the classical categories of stem and leaf. Hence, process morphology supersedes the strictures of classical morphology that is based on an either/or logic. Process combinations may change during evolution and shift their positions, leading to heterotopy.

For updates to this paper see Sattler, R. “Plant Evo-Devo of the Gynoecium: Heterotopy, Redefinition of the Carpel, and a Topographic Approach”. https://beyondwilber.ca/about/plant_evo-devo/gynoecium.html (accessed on 19 February 2024).

## Data Availability

Not applicable.
